# Doppler Ultrasound Triggering for Cardiovascular MRI at 3T in a Healthy Volunteer Study

**DOI:** 10.2463/mrms.mp.2015-0104

**Published:** 2016-03-21

**Authors:** Fabian Kording, Jin Yamamura, Gunnar Lund, Friedrich Ueberle, Caroline Jung, Gerhard Adam, Bjoern Philip Schoennagel

**Affiliations:** 1 University Medical Center Hamburg-Eppendorf, Center for Radiology and Endoscopy, Department of Diagnostic and Interventional Radiology, University Medical Center Hamburg-Eppendorf, Martinistraße 52, 20246 Hamburg, Germany; 2 Hamburg University of Applied Sciences, Faculty of Life Sciences

**Keywords:** cardiovascular magnetic resonance imaging, Doppler ultrasound, cardiac triggering

## Abstract

**Purpose:**

Electrocardiogram (ECG) triggering for cardiac magnetic resonance (CMR) may be influenced by electromagnetic interferences with increasing magnetic field strength. The aim of this study was to evaluate the performance of Doppler ultrasound (DUS) as an alternative trigger technique for CMR in comparison to ECG and pulse oximetry (POX) at 3T and using different sequence types.

**Methods:**

Balanced turbo field echo two-dimensional (2D) short axis cine CMR and 2D phase-contrast angiography of the ascending aorta was performed in 11 healthy volunteers at 3T using ECG, DUS, and POX for cardiac triggering. DUS and POX triggering were compared to the reference standard of ECG in terms of trigger quality (trigger detection and temporal variability), image quality [endocardial blurring (EB)], and functional measurements [left ventricular (LV) volumetry and aortic blood flow velocimetry].

**Results:**

Trigger signal detection and temporal variability did not differ significantly between ECG/DUS (I = 0.6) and ECG/POX (*P* = 0.4). Averaged EB was similar for ECG, DUS, and POX (p_ECG/DUS_ = 0.4, p_ECG/POX_ = 0.9). Diastolic EB was significantly decreased for DUS in comparison to ECG (*P* = 0.02) and POX (*P* = 0.04). The LV function assessment and aortic blood flow were not significantly different.

**Conclusion:**

This study demonstrated the feasibility of DUS for gating human CMR at 3T. The magnetohydrodynamic effect did not significantly disturb ECG triggering in this small healthy volunteer study. DUS showed a significant improvement in diastolic EB but could not be identified as a superior trigger method. The potential benefit of DUS has to be evaluated in a larger clinical patient population.

## Introduction

Cardiac magnetic resonance (CMR) imaging is increasingly performed at 3T with the merit of an approximately two-fold increase of the signal-to-noise ratio (SNR) compared to 1.5T.^1–3^ The improved image quality and spatial resolution implicate higher diagnostic accuracy with special benefit, e.g., performing myocardial perfusion imaging.^4^ A prerequisite to guarantee high-quality CMR images is an adequate synchronization of image acquisition with the cardiac cycle, which is usually achieved using the spatial information of an electrocardiogram (ECG). However, ECG signal disturbances with incorrect trigger are reported to occur in up to 35% of patients undergoing high-field CMR.^5^ With higher magnetic field strengths distortion of ECG signals with incorrect R-wave detection are mainly caused by gradients, radiofrequency (RF)-pulses, and also the magnetohydrodynamic (MHD) effect that occurs when a conductive fluid such as blood travels through a magnetic field. This flow-induced voltage is perpendicular to the magnetic field and blood flow direction and superimposes the ECG signal.^6,7^ Hence, quality of the ECG signal at 3T is generally decreased compared to 1.5T.^8^ As the MHD effect increases with the magnetic field strength, more complex algorithms are necessary to calculate a precise trigger for cardiac synchronization.^8–11^ However, the need for an increased complexity is prone to errors and may not be applicable to patients with cardiac pathologies.^9^ Incorrect R-wave recognition at higher field strength arises from the electrophysiological nature of the ECG signal and a method to synchronize the cardiac cycle with magnetic resonance imaging (MRI) acquisition that is not affected by the MHD effect would be desirable.

Strategies that are not affected by the MHD effect include techniques such as pulse oximetry (POX), self-gating, and acoustic phonocardiography.^5,12–17^

An additional trigger method which is theoretically not influenced by electromagnetic interferences was proposed by Rubin et al.^18^ in a pioneering work based on Doppler ultrasound (DUS). However, insufficient radiofrequency shielding and low SNR were identified as major drawbacks. In addition, simultaneously acquired ultrasound images have been used in several CMR applications for motion and organ tracking,^19–22^ but have not been used as a trigger source for CMR. In a more recent study, it was shown that DUS can be used as a trigger source for CMR with high accuracy, no artifacts, and a similar image and trigger quality compared to ECG.^23^ However, the performance of DUS was only evaluated at 1.5T and the feasibility and quality in comparison to standard methods such as the ECG cannot be easily transferred to higher field strength. More precisely, gradients, RF pulses, and the MHD effect may cause increased disturbances of trigger signals and the value of DUS has to be evaluated compared to 1.5T. Moreover, as alternative trigger techniques such as phonocardiogram gating cannot reliably be employed for different CMR techniques,^24^ the applicability of the DUS trigger source has to be evaluated for different sequence types.

Hence, the aim of this work was to compare the performance of DUS to a trigger technique, which is prone to the MHD effect such as ECG and to a technique that is not influenced by the MHD effect such as POX using different applications of CMR at 3T.

## Materials and Methods

### Study population

The study population to compare ECG, DUS, and POX-triggering consisted of 11 healthy male volunteers (mean age 28 ± 4 years) with no history of cardiovascular disease. In another three healthy volunteers, the performance of ECG and DUS triggering was tested performing whole heart coronary MR angiography (CMRA). The study was approved by the local ethics committee. Prior to MR examination informed written consent was obtained from each volunteer.

### DUS triggering

Cardiac triggering with DUS was realized with a modified commercially available cardiotocogram (CTG) (HP 8040A, Hewlett Packard, Palo Alto, CA, USA) as previously described.^23^

The CTG was placed outside of the scanner room and connected to the DUS transducer with a dedicated shielded CTG cable. A sufficient MR compatibility of the transducer was achieved by replacing all magnetic components of the DUS transducer.^25^ The transducer transmits 1.024 MHz ultrasound signals with a repetition frequency of 3.2 kHz, which are then processed by the CTG. Fetal cardiac examinations using the CTG usually do not provide an output for the raw DUS signal. Hence, the CTG was modified to access band pass filtered (0–10 Hz) raw DUS signals, which were transferred to a laptop using a data acquisition card (myDAQ, National Instruments, Texas, USA) with a sampling rate of 200 Hz.

The DUS transducer was placed in an apical position under the 32-element phased-array coil to obtain the DUS signal of transmitral flow that is characterized by two peaks, the E- and A-wave, respectively. The E-wave reflects early diastolic filling due to left ventricular (LV) relaxation whereas the A-wave represents late diastolic filling due to atrial contraction. The acoustic DUS signal of the CTG was used to indicate correct positioning of the transducer. If necessary, its location was varied until the characteristic E- and A-waves were clearly detectable. A dedicated peak detection algorithm implemented in LabView (National Instruments, Texas, USA) was used to select the E-wave as trigger time point and to generate a digital trigger signal.^23^ The trigger signal was then transferred to the physiologic interface of the MRI using a coaxial cable.

### Trigger evaluation

**Fig 1.**
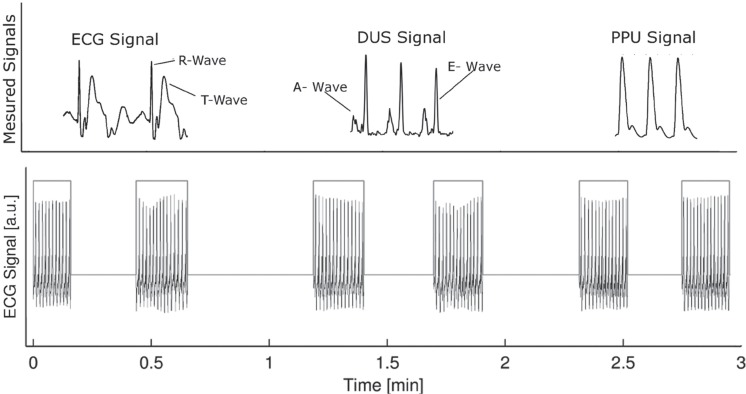
Example of recorded trigger signals. Top: The trigger signals for electrocardiogram (ECG), Doppler ultrasound (DUS), and pulse oximetry (POX) recorded by the log file with observed T-wave elevation in the ECG. Bottom: Only trigger signals that occurred during data acquisition and breath-hold were analyzed by the logging function of the magnetic resonance scanner.

The external trigger input of the physiologic unit of the MR scanner for DUS triggering enabled synchronous recording of ECG, DUS, and POX trigger signals. The logging function of the MRI provides an acquisition window that allows the selection of time periods of MR data collection during breath hold. For direct comparison between image quality and trigger quality, trigger signals during free breathing and absent MR data acquisition were excluded from trigger evaluation (Fig. 1). Hence, the trigger quality of DUS and POX signals could be directly compared to the reference standard of ECG in terms of RR interval length and temporal variability (temporal RR interval variability). The sensitivity of trigger detection was determined by manual confirmation of recorded QRS complexes and comparison with ECG, DUS, and POX trigger signals, respectively. In addition, simultaneous acquisition of trigger signals allowed evaluation of the occurrence of DUS and POX trigger signals in relation to the diastolic quiescent heart phase. Diastolic cardiac quiescence refers to an interval of the cardiac cycle with relatively stationary myocardium and insignificant wall motion and only marginal ventricular volume change,^26^ and is thus an important heart phase for imaging. The time delay of cardiac quiescence in relation to the R-wave was determined by visually defining the beginning of a motion-free cardiac wall in 2D cine CMR long axis views by two observers using commercial software (Extended MR Workspace, Philips Medical Systems, Best, The Netherlands). The determined time delay was then compared to the occurrence of DUS and POX trigger signals in the associated log file. As the occurrence of quiescent heart phases varies with the individual RR interval length, each time delay was normalized and expressed in percentage of the related RR interval.

### Data acquisition

CMR was performed at 3T (Ingenia, Philips Medical Systems) with the patient in supine position using the dedicated 32-element body coil. Scout images were performed in axial, coronal, and sagittal orientations. The ECG, DUS, and POX triggered breath-hold gradient echo 2D cine balanced turbo field echo (BTFE) sequences [matrix: 352 × 352, pixel spacing: 0.99 × 0.99 mm^2^, repetition time/echo time (TR/TE): 4.8/1.4 msec, FA: 45°, turbo factor: 13, SENSE: 2, slices: 14, slice thickness: 8 mm] encompassing the entire ventricle were acquired in short axis for LV function analysis with 30 cardiac phases resulting in an average temporal resolution of 30 msec. Moreover, DUS triggering for a different CMR technique was evaluated in 9 of the 11 examined subjects and compared to ECG and POX triggered CMR performing breath-hold 2D cine phase-contrast (PC) angiography sequences (matrix: 288 × 288, resolution: 1.2 × 1.2 mm TR/TE: 4.2/2.6 msec, FA: 10°, slice thickness: 8 mm, velocity encoding: 160 cm/s, phases: 25). Mean velocities, peak velocities, and stroke volume (SV) were assessed in the ascending aorta at the level of the pulmonary trunk. The sequence of the applied trigger method was alternated between subjects to render the influence of patient compliance which may vary with increasing scan time.

For CMRA, a navigator-gated free breathing 3D TFE gradient echo whole heart sequence was applied using ECG and DUS as trigger source [matrix: 480 × 480 × 105, resolution: 0.7 × 0.7 × 1 mm, TR/TE: 4.8 / 1.4 ms, flip angle (FA): 20°, slices: 53, fat saturation: SPIR, SENSE: 2].

### Image analysis

Image quality of all three trigger methods was directly assessed using the acutance between LV blood and myocardium.^23^ This method is based on the edge spread function of myocardial and ventricular signal intensities which allows calculation of endocardial border sharpness (EBS). For optimal image contrast, e.g., between black and white, two pixels are required whereas the slope of the edge spread function would be equal to one. However, ideal image contrast is distorted by noise generated by the MR system. More importantly, inaccurate synchronization of the heart cycle leads to image blurring which results in a reduced slope in the edge spread function. To assess an EBS impaired by motion blurring the slope of the edge spread function between myocardium and ventricular blood was calculated as

$$\text{EBS}=\frac{\text{max}(ds(r))}{dr},$$

where *S*(*r*) is the edge spread function. Prior to calculation, *S*(*r*) was normalized using mean ventricular signal intensities and baseline corrected by subtracting *S*(*r*) from mean myocardial signal intensities. Hence, 1/EBS determines the width of pixel between mean myocardial signal intensities and ventricular blood, and therefore describes endocardial blurring (EB).

The EB was calculated for mid-cardial and basal segments (with myocardial circumference >90%) using a dedicated algorithm in Matlab (The Mathworks, Natick, MA, USA).^23^ The ventricle was segmented into 36 radial sections for each slice and EB was calculated for each heart phase and section propagated over a whole RR interval. The cardiac cycle was divided into systole and diastole to calculate the respective systolic and diastolic EB for each trigger method. As the systole is a relatively constant time interval, the threshold defining the end of systole was set to 350 msec.^27^ Moreover, EB was calculated along the frequency and phase encoding direction.

The LV volumetry was assessed by a radiologist with 6 years’ experience in CMR. LV end-diastolic volumes (EDVs), end-systolic volumes (ESVs), SVs, and ejection fraction (EF) were determined by manual delineation of the endocardial borders in end-systolic and end-diastolic short axis views using a dedicated workstation (Extended MR Workspace, Philips Medical Systems). The most basal slice at the level of the mitral valve needed to include at least 50% of myocardial circumference to be included into volumetric analysis.^28^

For quantitative analysis of PC-MRI, the aortic lumen was segmented by manually drawing a region of interest (ROI) encompassing the cross-sectional vessel area on one single frame in the magnitude images. The missing contours for the remaining frames were generated automatically and ROIs were super-imposed to phase images by the software. The automated segmentation was followed by inspection and, if necessary, by manual correction for exclusion of the vessel wall. The SV determined by PC-MRI was calculated by integration of the area under the antegrade flow curve. The time-velocity curve was automatically calculated by the software tool.

### Statistical analysis

Data are presented as mean values ± standard deviation (SD). Potential differences of ECG, DUS, and POX trigger methods were evaluated using a one-way analysis of variance (ANOVA) for multiple groups in conjunction with Tukey’s honest significant difference (HSD) test. Differences within groups were evaluated using a paired t-test. The level of agreement and bias between methods were assessed using the method of Bland-Altman within a 95% confidence interval (CI).^29^ Inter-observer variability was measured using Kendall’s coefficient W of concordance, whereas 0 describes no agreement and 1 full agreement. All measurements were calculated using Matlab (Matlab, The Mathworks). A *P* value <0.05 was considered to indicate statistical significance. The *P* values are marked in figures according to the following criteria: ***P < 0.001; **P < 0.01; *P < 0.05.

## Results

### Trigger analysis

Synchronous recording of the three different trigger methods was successful in all subjects. The positioning of the DUS transducer on the volunteers’ chests took about 30 sec for each subject. The DUS transducer had to be repositioned during MR examination in one subject due to movement and corresponding loss of trigger signal. None of the volunteers experienced any discomfort due to the DUS sensor. Although ECG was affected by the MHD effect causing T-wave elevations in 8 from 11 subjects (Fig. 1), CMR acquisition using ECG triggering could be conducted in all subjects.

**Fig 2.**
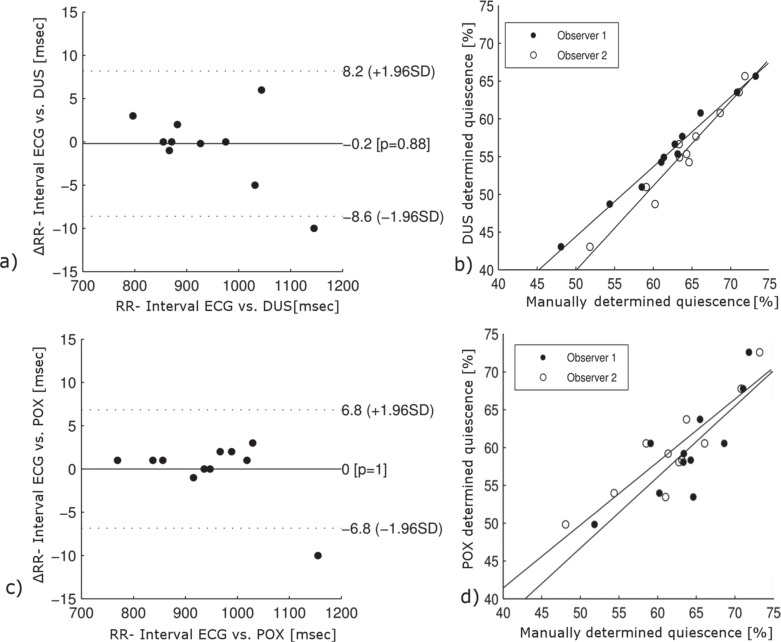
Analysis of simultaneous acquired trigger signals. Bland-Altman plot of mean RR intervals and trigger signal intervals determined by (**a**) electrocardiogram (ECG) and Doppler ultrasound (DUS) and (**c**) ECG and pulse oximetry (POX). Dashed lines represent the confidence interval of ±1.96 of the standard deviation. Correlation between visually determined quiescent diastolic heart phases and time between R-wave and trigger signals of (**b**) DUS and (**d**) POX.

The mean RR interval was 926 ± 111 msec. Results for each trigger method are shown in Table 1. Simultaneously recorded DUS and POX trigger signals strongly correlated with recorded ECG traces (R = 0.9 and R = 0.9) with a mean difference to ECG of −0.2 ± 0.4 msec [CI: (−2.9 to 2.9 msec)] (DUS) and −0.3 ± −0.5 msec [CI: (−4.4 to 3.9 msec)] (POX) (Fig. 2). The sensitivity of heart cycle recognition referring to ECG (99 ± 1%) was not significantly different for DUS (99 ± 1%, *P* = 0.6) and POX (99 ± 1%, *P* = 0.4) triggered acquisitions. Likewise, no significant difference was obtained for the variability between RR intervals for ECG (32 ± 12 msec), DUS (27 ± 10 msec, *P* = 0.8), and POX (27 ± 10 msec, *P* = 0.9). The delay in trigger recognition was not different (*P* = 0.9) between ECG (6 ± 3 msec) and DUS (4 ± 0.4 msec) but significantly increased for POX (49 ± 9 msec) compared to ECG (*P* < 0.0001).

Quiescent diastolic heart phases occurred at 62 ± 6% (observer 1) and 63 ± 5% (observer 2) of the RR interval using ECG triggering. Inter-observer agreement for determination of cardiac quiescence was very high (R = 0.9, W = 0.93). The DUS trigger signals occurred at 55 ± 6% of the RR interval and were strongly correlated to quiescent heart phases determined by observer 1 (R = 0.9) and observer 2 (R = 0.9). The POX trigger signals occurred at 60 ± 6% of the RR interval and were also strongly correlated to quiescent heart phases determined by observer 1 (R = 0.9) and observer 2 (R = 0.9) (Fig. 2). The DUS signal appeared 6 ± 1%/8 ± 1% earlier, and the POX signal 2 ± 3%/4 ± 4% earlier than the visually determined beginning of the quiescent heart phases measured by observer 1 and 2, respectively.

### Functional parameters

**Fig 3.**
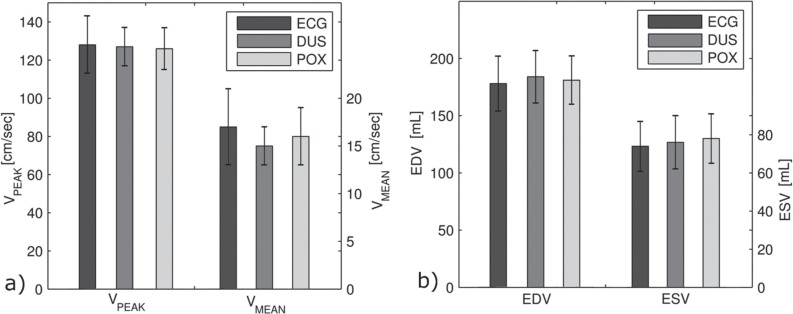
Mean results of LV function analysis and velocimetry. Mean values (±standard deviation) of parameters assessed from velocimetry in the ascending aorta (**a**) and LV volumetry (**b**) for each trigger method. DUS, Doppler ultrasound; ECG, electrocardiogram; EDV, end diastolic volume; ESV, end systolic volume; LV, left ventricular; POX, pulse oximetry; V_mean_, mean velocity; V_peak_, peak velocity.

**Fig 4.**
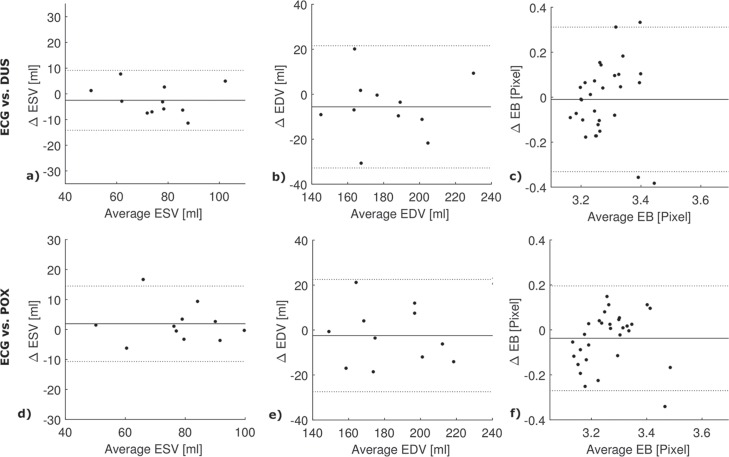
Bland-Altman plots in reference to the electrocardiogram. Bland-Altman plots of end-diastolic volumes (EDVs), end-systolic volumes (ESVs) and endocardial blurring (EB) are shown in reference to the ECG for DUS (**a**–**c**) and for POX (**d**–**f**). Dashed lines represent the confidence interval of ±1.96 of the standard deviation.

**Fig 5.**
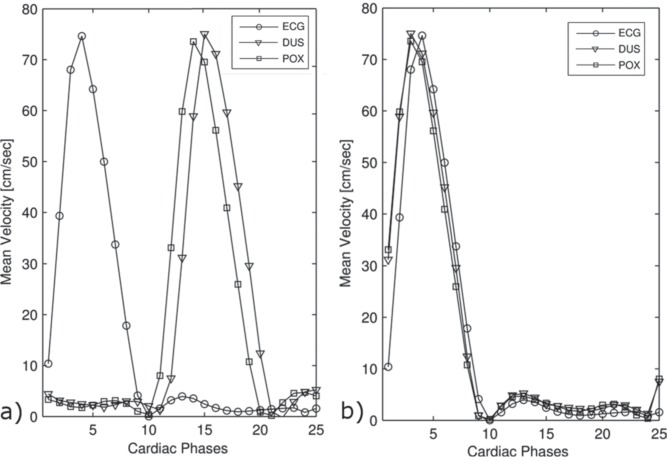
Aortic blood flow velocimetry based on different trigger methods. (**a**) Time velocity curves (mean velocities) averaged over all subjects for each trigger method. A time shift of the averaged curves is illustrated according to different trigger time points of ECG, DUS, and POX. The resampled time velocity curves referring to the trigger time delay of DUS and POX in comparison to ECG are shown in (**b**). DUS, Doppler ultrasound; ECG, electrocardiogram; POX, pulse oximetry.

Parameters assessed from LV volumetry and aortic velocimetry did not significantly differ between trigger methods (Table 2). For ECG, DUS, and POX triggered 2D cine short axis BTFE sequence mean EDV was 178 ± 24 ml, 184 ± 24 ml, and 181 ± 22 ml; mean ESV was 74 ± 13 ml, 76 ±14 ml, and 78 ± 14 ml. Resulting SV was 104 ± 13 ml, 107 ± 12 ml, and 103 ± 11 ml; EF was 58 ± 4%, 58 ± 4%, and 57 ± 4% (Fig. 3). The mean difference for EDV in reference to ECG was −2 ± 12 ml [CI: (−11 to 6 ml)] for DUS, −5 ± 13 ml [CI: (−14 to 21 ml)] for POX. For ESV, the mean difference was 2 ± 6 ml [CI: (−2 to 6 ml)] for DUS and −3 ± 6 [CI: (−7 to 1 ml)] for POX (Fig. 4). As the time velocity curves of DUS and POX are right-shifted due to later trigger time points, the curves averaged over all subjects were resampled according to the mean time delay of the trigger in respect to ECG (Fig. 5). For ECG, DUS, and POX triggered 2D cine PC angiography SV was 116 ± 13 ml, 110 ± 13 ml, and 113 ± 13. Average peak velocities (V_Peak_) and mean velocities (V_Mean_) did not significantly differ with V_Peak_ = 128 ± 14 cm/sec, 127 ± 10 cm/sec, 126 ± 11 cm/sec, and V_Mean_ = 16 ± 4 cm/sec, 15 ± 2 cm/sec, and 16 ± 3 cm/sec, respectively.

### Image quality

**Fig 6.**
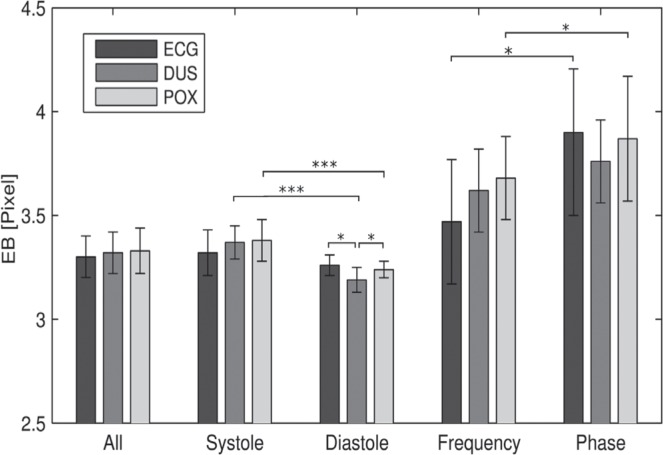
Endocardial blurring (EB) for each trigger method. Mean EB (± standard deviation) for electrocardiogram (ECG), Doppler ultrasound (DUS), and pulse oximetry (POX) triggering averaged over the entire cardiac cycle (All), referring to the cardiac cycle (diastole and systole) and encoding direction (frequency and phase). Significant differences between cardiac cycles, encoding direction, and trigger methods are marked within the figure.

**Fig 7.**
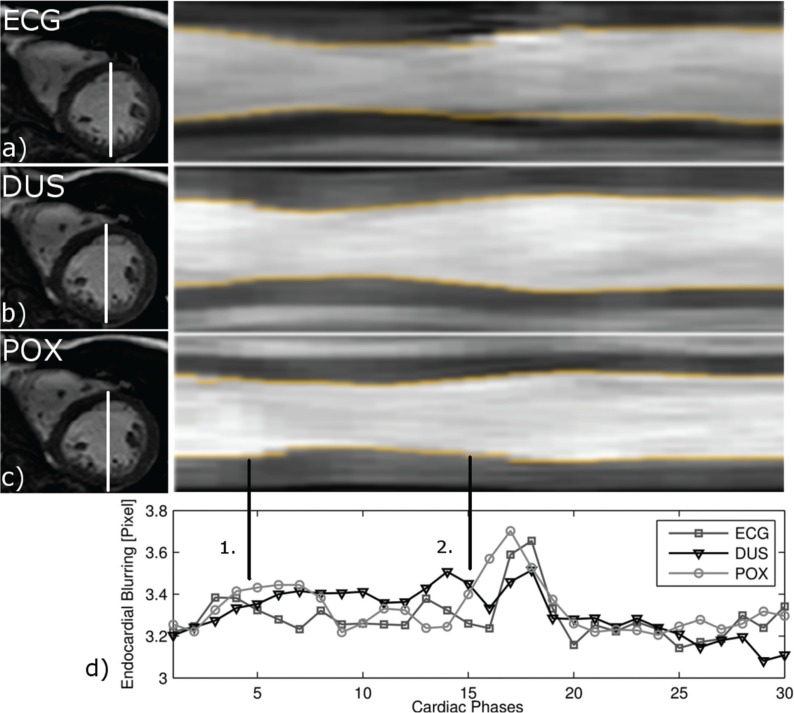
Left ventricular (LV) myocardial wall motion and endocardial blurring (EB) for each trigger method. Mid-ventricular short-axis images in end diastole for each trigger method [left column of (**a**–**c**)] and corresponding projection of LV myocardial wall motion over the cardiac cycle (time-motion equivalent) with detected endocardial blurring (EB) (**d**) at the endocardial border (yellow). The images and EB were resampled using the delay of the trigger with respect to the electrocardiogram. Average EB for each trigger method over the cardiac cycle is shown in the bottom row. EB is sensitive to cardiac phases (1. = systole, 2. = diastole), revealing a peak during early diastole (phases 16–19) and flattened plateau-like shape during later diastole. ECG, electrocardiogram; DUS, Doppler ultrasound; POX, pulse oximetry.

The border sharpness between ventricular blood and myocardium was successfully determined for each trigger method. An average of 5 slices was included for analysis resulting in 5400 sample points per subject. Mean EB averaged over the entire cardiac cycle was similar for ECG, DUS, and POX (3.30 ± 0.12 pixel, 3.33 ± 0.14 pixel, 3.33 ± 0.11 pixel) with no significant differences (p_ECG/DUS_ = 0.5; p_ECG/POX_ = 0.9; p_DUS/POX_ = 0.6). The mean difference in reference to ECG for DUS was −0.03 ± 0.11 [CI: (−0.08 to 0.01 pixel] and for POX −0.01 ± 0.16 [CI: (−0.07 to 0.05 pixel)]. Significant differences were obtained between systolic and diastolic EB for DUS (3.37 ± 0.05 pixel and 3.19 ± 0.06 pixel; *P* < 0.0001) and POX (3.38 ± 0.10 pixel and 3.24 ± 0.04 pixel; *P* = 0.0003), but not for ECG (3.32 ± 0.11 pixel and 3.26 ± 0.05 pixel; *P* = 0.06). Systolic EB was not significant different between trigger methods (p_ECG/DUS_ = 0.2; p_ECG/POX_ = 0.9; p_DUS/POX_ = 0.1) (Fig. 6). Instead, early diastolic EB was significantly reduced for DUS compared to ECG (p_ECG/DUS_ = 0.02) and POX (p_DUS/POX_ = 0.04), but not for ECG versus POX (p_ECG/POX_ = 0.9) (Fig. 7). The EB was significantly increased in the phase encoding direction compared to the frequency encoding direction for ECG (3.90 ± 0.4 pixel vs. 3.47 ± 0.36 pixel; *P* = 0.01) and POX (3.87 ± 0.4 pixel vs. 3.68 ± 0.2 pixel; *P* = 0.01), but not for DUS (3.76 ± 0.2 pixel vs. 3.62 ± 0.2 pixel; *P* = 0.11) (Fig. 6).

**Fig 8.**
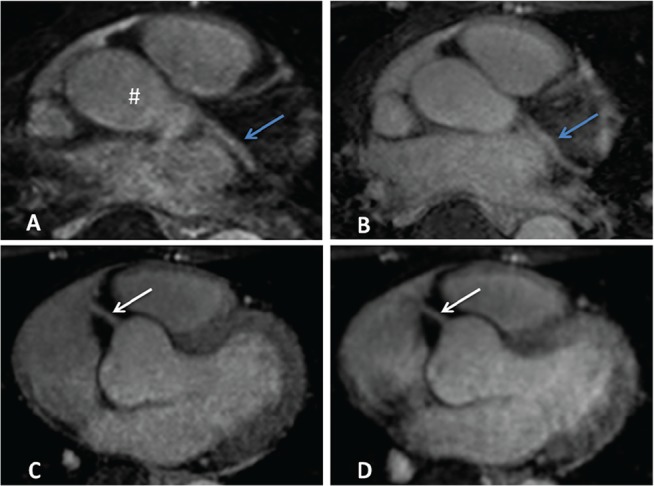
ECG and DUS triggered exemplary coronary magnetic resonance angiography images. Transversal images of the navigator-gated free breathing three-dimensional turbo field echo gradient echo whole heart sequence using ECG (**B**, **D**) and DUS (**A**, **C**) triggering. Similar image quality allowed accurate delineation of the left main coronary artery branches (**A**, **B**) with ascending aorta (#) and left circumflex artery (blue arrows) and the proximal right coronary artery (**C**, **D**) (white arrows). ECG, electrocardiogram; DUS, Doppler ultrasound.

In the pilot approach performing CMRA using ECG and DUS triggering similar image quality was achieved by visual assessment (Fig. 8). As DUS trigger signals occur at 55 ± 60% of the ECG RR interval, representing the quiescent diastolic heart phase, no manual selection to start image acquisition was necessary using DUS.

## Discussion

Our study demonstrated the feasibility of DUS as an alternative trigger technique for human CMR at 3T. The DUS-triggered CMR using different sequence types was successful in all subjects and revealed similar results in terms of accurate trigger quality, LV volumetry, aortic velocimetry, and image quality compared to ECG and POX triggered acquisitions. Despite distortion in the ECG due to the MHD effect, the trigger methods were not significantly different and showed a high level of agreement. An increased image quality could be achieved using DUS by a decrease in EB during early and the very late diastole compared to ECG and POX. With the increasing application of 3T CMR and possible distortions of ECG trigger signals that may influence image quality, our results emphasize the potential of DUS as an alternative CMR trigger method. As the trigger signal is generated during early diastole, DUS gating could be of interest in special fields of applications such as MR coronary artery angiography where ECG gating still requires manual adjustment of trigger delay due to inter-individual heart rate variability.

In this work, the DUS transducer was placed under the body coil during CMR where the maximum of interference and associated distortions are expected. The CMR acquisition was successful in all volunteers without image artifacts, MR signal loss, or interferences in the DUS signal. In comparison, the ECG may be disturbed by the MHD effect, RF pulses, or gradient artifacts, which overlap with the bandwidth of the ECG signal and cannot be removed by simple low-pass filtering.^10^ More complex methods have to be used to address image disturbances caused by MR-related noise, the MHD effect, and arrhythmias. Existing methods are either able to reject arrhythmias or the MHD effect: adaptive noise cancelling achieved good results to reject arrhythmias but not addressing the MHD effect.^10^ In contrast, a 12-lead ECG for triggering high field CMR failed for an arrhythmia rejection.^30^ The spatial information of an ECG does not allow arrhythmia rejection in cases of atrial fibrillation with ectopic intervals as no change in the QRS loop occurs.^8,11^ In contrast, DUS is not affected by the MHD effect. As DUS signals are related to transmittal flow instead of electrophysiological activation (QRS) it thus theoretically allows the rejection of ectopic QRS-appearance. Moreover, it can be used for fetal CMR where triggering using conventional methods is not feasible.^25,31^ Acoustic triggering based on a phonocardiogram or POX has been successfully used for cine acquisitions in high-field CMR as an alternative trigger method.^12,15^ However, switching gradients introduce noise to the acoustic triggering device resulting in limited reliability for PC angiography acquisitions and thus suggesting dependency on sequence type.^24^

For a direct comparison and evaluation of ECG, DUS, and POX, trigger signals were recorded simultaneously during CMR acquisition. DUS and POX recorded cardiac cycle lengths revealed very good agreement with the reference standard of ECG in our study. The 99% sensitivity of trigger detection for each method is in accordance with results for an ECG using spatial information^11^ and DUS.^23^ However, R-wave mis-registration with resulting cardiac motion artifacts was reported to occur in up to 30% at 7T^15^ and even hampered CMR examination in 35% of patients at 3T.^5^ Although the MHD effect was present in 72% of all examinations (Fig. 1), the trigger quality of ECG was not significantly different compared to DUS and POX. The observed variability for ECG, DUS, and POX were in the range of reported ECG heart rate variability of 23 ± 12 msec in healthy controls,^32^ and small in comparison to previously reported values for POX of 65 msec at 7T^15^ and 82 msec at 1.5T,^23^ that may limit reliable synchronization. The temporal variability is an important measure as retrospective cine sequences distribute the image acquisition over multiple intervals. Thus, a large variability leads to motion artifacts, blurring, and impaired image quality.^9^ However, the variability is rather an indicator than a qualitative measure as heart rate variability is not considered.

The DUS trigger signal originates from the E-wave during the rapid LV filling and hence occurs in early- to mid-diastole.^33^ The POX signal measures the change in absorption rate of infrared light during systole and diastole and corresponds to the absorption caused by arterialized blood.^34^ The quiescent heart phase with minimal cardiac wall motion was determined to begin at 63% of the RR interval, resembling mid-diastole. As DUS and POX trigger signals occurred at 56% and 60%, respectively, these trigger signals may be an appropriate method to depict cardiac quiescence. We found a strong correlation between the beginning of cardiac quiescence and DUS and POX trigger signals with DUS and POX signals occurring 6% and 2% earlier, respectively. This may be of particular interest in coronary artery imaging where a biphasic pattern of coronary artery motion during the cardiac cycle is present, with an optimal scan time due to cardiac rest period during end-systole and also mid-diastole (60–80%).^35,36^ To date, the variability of POX measurements to define cardiac quiescence was higher compared to DUS and in the range of the temporal resolution (≈30 msec), suggesting that precise identification of quiescent heart phases is more reliable using DUS. In the first approach, the feasibility to use DUS for CMRA in conjunction with an automatic selection of diastolic cardiac quiescence was successfully evaluated in comparison to ECG. However, a possible improvement for CMRA using DUS with a detailed analysis of the image quality needs further evaluation in a larger patient population.

Comparing the three applied trigger methods, no significant differences were found for LV volumetry and aortic velocimetry. All assessed parameters were in the range of reported inter-study variability.^37,38^

The observed wavelike increase of diastolic EB during atrial contraction in this study can be explained by peak radial and longitudinal velocities of the mid-ventricular and basal myocardium at this phase of the cardiac cycle.^39^ A significantly decreased EB was observed for DUS triggering during this phase of the cardiac cycle. The following decrease of EB during diastole, irrespective of the trigger method, corresponds with cardiac quiescence due to reduced radial, tangential, and longitudinal myocardial velocities.^39^ The findings of decreased diastolic EB using DUS may be explained by the different trigger time points of ECG, DUS, and POX. A constant RR interval length is assumed for segmented cine acquisitions, however, the interval length changes due to intra-individual heart rate variability. Hence, blurring effects may be decreased for heart phases close to the trigger time point and, inversely, increase with later heart phases. This hypothesis is emphasized by the fact that systolic EB using ECG is decreased compared to DUS- and POX-triggering. However, the assumed effect of increasing EB with increasing heart phase is small and does not affect functional analysis. Although the POX trigger occurs only shortly (40–60 msec) after the DUS trigger, no decrease of diastolic EB was apparent. This may be due to the broad shape of the POX signal where the peak is more difficult to define^5^ and thus revealed higher variations in cardiac cycle length and therefore increased EB compared to DUS.

The EB calculation was sufficient to reveal the expected differences of EB in frequency and phase encoding direction, as motion-related blurring occurs in the phase encoding direction.^40^

A limitation of DUS application is patient compliance as motion can affect the position of the transducer. In addition, as the DUS trigger refers to the E-wave, its detection could be influenced in cases such as mitral valve insufficiency, severe left heart failure, diastolic dysfunction, or tachycardia. Hence, the signal quality of DUS for cardiac triggering may be distorted due to pathologic conditions and needs further evaluation in future studies. Although DUS itself is not influenced by RF transmit fields or eddy currents, it is still based on the measurement of a voltage across piezo elements and requires conducting wires which may interact with the MRI. However, possible distortions in the DUS signal between the 1 MHz ultrasound signal and induced RF signals of 127 MHz are well delimited and hence preventable using simple low-pass filtering. Finally, the small number of examined volunteers may not allow drawing general conclusions. The reported influence of the MHD effect on ECG triggering by Sievers et al.^5^ could not be verified in this study. One reason may be the small study group with only healthy volunteers. Therefore, DUS application needs to be evaluated in a clinical setting with larger patient numbers, e.g., in patients with arrhythmias or mitral valve defects, to confirm theoretical benefits.

## Conclusion

In conclusion, DUS proved to be a reliable gating method for CMR at 3T using different sequence types. DUS gating revealed no significant differences compared to ECG and POX in terms of trigger quality, image quality, and functional measurements but showed a significant improvement in EB during diastole. Future studies have to investigate the potential benefits of DUS in a patient population with cardiac pathologies and at higher field strength where the MHD effect is even more prominent.

## Figures and Tables

**Table 1.** Comparison of cardiac cycle lengths and trigger detection

	ECG	DUS	POX
RR intervals	82 ± 13	82 ± 11	84 ± 11
RR length (msec)	926 ± 111	926 ± 111	929 ± 111
Difference in RR length (msec)	-	−0.2 ± 0.4	0.3 ± 0.5
Variability (ms)	31 ± 12	27 ± 10	26 ± 8
Sensitivity (%)	99 ± 1	99 ± 1	99 ± 2
ΔT (ms)	6 ± 3	4 ± 0.4	49 ± 9

Lengths of cardiac cycles (R-wave to R-Wave) for all subjects according to trigger methods with mean differences in respect to ECG. Total count of cardiac intervals for each cine acquisition with corresponding sensitivities for each trigger method and time delay of trigger recognition (ΔT). DUS, Doppler ultrasound; ECG, electrocardiogram; POX, pulse oximetry; RR, cardiac cycle.

**Table 2.** Comparison of functional parameters with reference to the applied trigger method

	ECG vs. DUS		ECG vs. POX		Subjects
	
	Difference	*P* value	Difference	*P* value	
EDV(ml)	5 ± 13	0.9	3 ± 12	0.8	11
ESV (ml)	3 ± 6	0.9	−2 ± 6	0.9	11
EF (%)	−0.1 ± 1.8	0.6	2 ± 2	0.9	11
SV (ml)	3 ± 9	0.8	4 ± 8	0.9	11
SV_PC_ (ml)	−6 ± 4	0.8	−4 ± 7	0.6	9
V_mean_ (cm/sec)	−2 ± 2	0.8	−1 ± 2	0.5	9
V_peak_ (cm/sec)	0.5 ± 6.3	0.9	2 ± 6	0.9	9

Mean differences of parameters assessed from left ventrricular (LV) volumetry and velocimetry in the ascending aorta for each trigger method in respect to electrocardiogram (ECG). DUS, Doppler ultrasound; EDV, end diastolic volume; EF, ejection fraction; POX, pulse oximetry; ESV, end systolic volume; SV, stroke volume assessed by LV volumetry; SV_PC_, stroke volume assessed by velocimetry; V_mean_, mean velocity; V_peak_, peak velocity.

## References

[1] Cheng AS Selvanayagam JB. High field cardiac magnetic resonance imaging—current and future perspectives. Heart Lung Circ 2010; 19:145–153. 20144558 10.1016/j.hlc.2009.11.008

[2] Kelle S Hamdan A Schnackenburg B Dobutamine stress cardiovascular magnetic resonance at 3 Tesla. J Cardiovasc Magn Reson 2008; 10:44. 18844984 10.1186/1532-429X-10-44 PMC2572055

[3] Gutberlet M Schwinge K Freyhardt P Influence of high magnetic field strengths and parallel acquisition strategies on image quality in cardiac 2D CINE magnetic resonance imaging: comparison of 1.5 T vs. 3.0 T. Eur Radiol 2005; 15:1586–1597. 15875193 10.1007/s00330-005-2768-z

[4] Gerretsen S Versluis B Bekkers S Leiner T. Cardiac cine MRI: comparison of 1.5 T, non-enhanced 3.0 T and blood pool enhanced 3.0 T imaging. Eur J Radiol 2008; 65: 80–85. 18155867 10.1016/j.ejrad.2007.11.004

[5] Sievers B Wiesner M Kiria N Speiser U Schoen S Strasser RH. Influence of the trigger technique on ventricular function measurements using 3-Tesla magnetic resonance imaging: comparison of ECG versus pulse wave triggering. Acta Radiol 2011; 52:385–392. 21498278 10.1258/ar.2011.100505

[6] Nijm GM Swiryn S Larson AC Sahakian AV. Extraction of the magnetohydrodynamic blood flow potential from the surface electrocardiogram in magnetic resonance imaging. Med Biol Eng Comput 2008; 46:729–733. 18239947 10.1007/s11517-008-0307-1

[7] Abi-Abdallah D Robin V Drochon A Fokapu O. Alterations in human ECG due to the Magnetoydro-Dynamic effect: a method for accurate R peak detection in the presence of high MHD artifacts. Conf Proc IEEE Eng Med Biol Soc 2007; 2007:1842–1845. 18002339 10.1109/IEMBS.2007.4352673

[8] Dietrich O Reiser MF Schoenberg SO. Artifacts in 3-T MRI: physical background and reduction strategies. Eur Radiol 2008; 65:29–35. 10.1016/j.ejrad.2007.11.005 18162353

[9] Krug JW Rose G Clifford GD Oster J. ECG-based gating in ultra high field cardiovascular magnetic resonance using an independent component analysis approach. J Cardiovasc Magn Reson 2013; 15:104. 24252594 10.1186/1532-429X-15-104 PMC4174900

[10] Wu V Barbash IM Ratnayaka K Adaptive noise cancellation to suppress electrocardiography artifacts during real-time interventional MRI. J Magn Reson Imaging 2011; 33:1184–1193. 21509878 10.1002/jmri.22530 PMC3080760

[11] Chia JM Fischer SE Wickline SA Lorenz CH. Performance of QRS detection for cardiac magnetic resonance imaging with a novel vectorcardiographic triggering method. J Magn Reson Imaging 2000; 12:678–688. 11050637 10.1002/1522-2586(200011)12:5<678::aid-jmri4>3.0.co;2-5

[12] Becker M Frauenrath T Hezel F Comparison of left ventricular function assessment using phonocardiogram- and electrocardiogram-triggered 2D SSFP CINE MR imaging at 1.5 T and 3.0 T. Eur Radiol 2010; 20:1344–1355. 20013275 10.1007/s00330-009-1676-z

[13] Crowe ME Larson AC Zhang Q Automated rectilinear self-gated cardiac cine imaging. Magn Reson in Med 2004; 52:782–788. 15389958 10.1002/mrm.20212

[14] Frauenrath T Hezel F Heinrichs U Feasibility of cardiac gating free of interference with electro-magnetic fields at 1.5 Tesla, 3.0 Tesla and 7.0 Tesla using an MR-stethoscope. Invest Radiol 2009; 44:539–547. 19652614 10.1097/RLI.0b013e3181b4c15e

[15] Frauenrath T Hezel F Renz W Acoustic cardiac triggering: a practical solution for synchronization and gating of cardiovascular magnetic resonance at 7 Tesla. J Cardiovasc Magn Reson 2010; 12:67. 21080933 10.1186/1532-429X-12-67 PMC2998500

[16] Larson AC White RD Laub G McVeigh ER Li D Simonetti OP. Self-gated cardiac cine MRI. Magn Reson in Med 2004; 51:93–102. 14705049 10.1002/mrm.10664 PMC2396326

[17] Usman M Atkinson D Kolbitsch C Schaeffter T Prieto C. Manifold learning based ECG-free free-breathing cardiac CINE MRI. J Magn Reson Imaging 2015; 41:1521–1527. 25124545 10.1002/jmri.24731

[18] Rubin JM Fowlkes JB Prince MR Rhee RT Chenevert TL. Doppler US gating of cardiac MR imaging. Acad Radiol 2000; 7:1116–1122. 11131056 10.1016/s1076-6332(00)80065-3

[19] Feinberg D Guenther M. Simultaneous MR and ultrasound imaging: towards US-navigated MRI. Proc Intl Soc Mag Reson Med 2003; 381.

[20] Feinberg DA Giese D Bongers DA Hybrid ultrasound MRI for improved cardiac imaging and real-time respiration control. Magn Reson Med 2010; 63:290–296. 20025068 10.1002/mrm.22250 PMC2813925

[21] Günther M Feinberg DA. Ultrasound-guided MRI: preliminary results using a motion phantom. Mag Reson Med 2004; 52:27–32. 10.1002/mrm.20140 15236363

[22] Petrusca L Cattin P De Luca V Hybrid ultrasound/magnetic resonance simultaneous acquisition and image fusion for motion monitoring in the upper abdomen. Invest Radiol 2013; 48:333–340. 23399812 10.1097/RLI.0b013e31828236c3

[23] Kording F Schoennagel B Lund G Doppler ultrasound compared with electrocardiogram and pulse oximetry cardiac triggering: a pilot study. Magn Reson Med 2015; 74:1257–165. 25359183 10.1002/mrm.25502

[24] Nassenstein K Orzada S Haering L Cardiac MRI: evaluation of phonocardiogram-gated cine imaging for the assessment of global und regional left ventricular function in clinical routine. Eur Radiol 2012; 22:559–568. 21947482 10.1007/s00330-011-2279-z

[25] Yamamura J Kopp I Frisch M Cardiac MRI of the fetal heart using a novel triggering method: initial results in an animal model. J Magn Reson Imaging 2012; 35: 1071–1076. 22246623 10.1002/jmri.23541

[26] Slavin GS Fung M. Electromechanical analysis of optimal trigger delays for cardiac MRI. J Cardiovasc Magn Reson 2014; 16:1–3.24387349

[27] Stuber M Botnar RM Danias PG Kissinger KV Manning WJ. Submillimeter three-dimensional coronary MR angiography with real-time navigator correction: comparison of navigator locations. Radiology 1999; 212: 579–587. 10429721 10.1148/radiology.212.2.r99au50579

[28] Alfakih K Plein S Thiele H Jones T Ridgway JP Sivananthan MU. Normal human left and right ventricular dimensions for MRI as assessed by turbo gradient echo and steady-state free precession imaging sequences. J Magn Reson Imaging 2003; 17:323–329. 12594722 10.1002/jmri.10262

[29] Bland JM Altman DG. Statistical methods for assessing agreement between two methods of clinical measurement. Lancet 1986; 1:307–310. 2868172

[30] Gregory TS Schmidt EJ Zhang SH Ho Tse ZT. 3DQRS: A method to obtain reliable QRS complex detection within high field MRI using 12-lead electrocardiogram traces. Magn Reson Med 2014; 71:1374–1380. 24453116 10.1002/mrm.25078 PMC3961533

[31] Schoennagel BP Remus CC Yamamura J Fetal blood flow velocimetry by phase-contrast MRI using a new triggering method and comparison with Doppler ultrasound in a sheep model: a pilot study. MAGMA 2014; 27:237–244. 23934159 10.1007/s10334-013-0397-0

[32] Mada RO Lysyansky P Daraban AM Duchenne J Voigt JU. How to define end-diastole and end-systole?: impact of timing on strain measurements. JACC Cardiovasc Imaging 2015; 8:148–157. 25577447 10.1016/j.jcmg.2014.10.010

[33] Jawad IA. A Practical Guide to Echocardiography and Cardiac Doppler Ultrasound. Lippincott Williams & Wilkins, 1996.

[34] Alexander CM Teller LE Gross JB. Principles of pulse oximetry: theoretical and practical considerations. Anesth Analg 1989; 68:368–376. 2645811

[35] Lu B Mao S-S Zhuang N Coronary artery motion during the cardiac cycle and optimal ECG triggering for coronary artery imaging. Invest Radiol 2001; 36:250–256. 11323512 10.1097/00004424-200105000-00002

[36] Shechter G Resar JR McVeigh ER. Rest period duration of the coronary arteries: implications for magnetic resonance coronary angiography. Med Phys 2005; 32:255–262. 15719976 10.1118/1.1836291 PMC2396325

[37] Hudsmith LE Petersen SE Francis JM Robson MD Neubauer S. Normal human left and right ventricular and left atrial dimensions using steady state free precession magnetic resonance imaging. J Cardiovasc Magn Reson 2005; 7:775–782. 16353438 10.1080/10976640500295516

[38] Maceira A Prasad S Khan M Pennell D. Normalized left ventricular systolic and diastolic function by steady state free precession cardiovascular magnetic resonance. J Cardiovasc Magn Reson 2006; 8:417–426. 16755827 10.1080/10976640600572889

[39] Jung B Markl M Föll D Hennig J. Investigating myocardial motion by MRI using tissue phase mapping. Eur J Cardiothorac Surg 2006; 29 Suppl 1:150–157. 16563784 10.1016/j.ejcts.2006.02.066

[40] Bogaert J Dymarkowski S Taylor AM Muthurangu V. Clinical Cardiac MRI. New York: Springer-Verlag Berlin Heidelberg, 2012.

